# Using Instagram to Promote Youth Mental Health: Feasibility and Acceptability of a Brief Social Contact-Based Video Intervention to Reduce Depression Stigma

**DOI:** 10.1192/j.eurpsy.2025.301

**Published:** 2025-08-26

**Authors:** D. Amsalem, S. Haim-Nachum

**Affiliations:** 1 Department of Psychiatry, Columbia University, New York, United States; 2 School of Social Work, Tel Aviv University, Tel Aviv, Israel

## Abstract

**Introduction:**

Depression is a leading cause of disability among youth, with stigma significantly hindering mental health service utilization. Untreated depression is associated with greater severity, poorer outcome, and cognitive impairment. In addition to structural barriers to service use, stigma towards mental health is a profound obstacle that impedes individuals from seeking needed services. Reducing stigma toward depression can improve perceptions of treatment and engage young individuals with depressive symptoms in behavioral services.

**Objectives:**

This study evaluates the feasibility and acceptability of delivering brief video interventions, proven to reduce depression stigma, via Instagram to promote help-seeking among youth. We hypothesized that the intervention would be both feasible and acceptable, generating higher link clicks and lower cost-per-click (CPC) compared to control videos.

**Methods:**

A two-week Instagram campaign in February 2024 targeted U.S. adolescents aged 14-22. The campaign featured a 60-second human-narrated personal story video, previously shown to reduce depression-related stigma. The video showcased a young individual sharing their experience with depression, misconceptions about treatment, and the support they received. Engagement metrics assessed included impressions (video displays), reach (distinct viewers), link clicks (engagement with mental health resources), and CPC (cost-effectiveness). These were compared to four control videos: AI-narrated personal story (“Control 1”), human-narrated educational text (“Control 2”), AI-narrated educational text (“Control 3”), and an un-narrated educational text with background music (“Control 4”).

**Results:**

All five videos successfully reached a broad audience, with a combined total of 808,000 impressions, 287,000 viewers, and 4,148 link clicks. The Human-Narrated Personal Story (Intervention) video was highly effective, achieving 874 link clicks and a competitive CPC of $0.92, outperforming three of the four control videos. The AI-Narrated Educational Text (Control 3) had the highest number of link clicks (985) and lowest CPC ($0.82), while the AI-Narrated Personal Story (Control 1) had the fewest link clicks (680) and highest CPC ($1.18).

**Conclusions:**

The intervention video successfully engaged the target audience, supporting its feasibility and acceptability for broader dissemination. Instagram proves to be a viable platform for disseminating evidence-based mental health interventions aimed at reducing depression stigma among youth. The study supports the potential for using social media in public mental health strategies, though further research is needed to evaluate its impact on help-seeking behaviors and across diverse demographic groups.

**Disclosure of Interest:**

None Declared

